# CCR9 shapes the immune microenvironment of colorectal cancer modulating the balance between intratumoral CD8^+^ T cell and FoxP3^+^ Helios^+^ Treg subpopulations

**DOI:** 10.1371/journal.pone.0321930

**Published:** 2025-04-30

**Authors:** Jacobo Martínez-Ríos, Cynthia Paola López-Pacheco, Eduardo Alberto García-Zepeda, Gloria Soldevila

**Affiliations:** 1 Departamento de Inmunología, Instituto de Investigaciones Biomédicas, Universidad Nacional Autónoma de México, Mexico City, Mexico; 2 Investigadora por México, Secretaría de Ciencia Tecnología y Humanidades (SECIHTI), Mexico City, Mexico; 3 Laboratorio Nacional de Citometría de Flujo, Instituto de Investigaciones Biomédicas, Universidad Nacional Autónoma de México, Mexico City, Mexico; Cincinnati Children's Hospital Medical Center, UNITED STATES OF AMERICA

## Abstract

Colorectal cancer (CRC) is the third most common cancer in the world and the second cause of death related to cancer. Regulatory T cell (Treg) infiltration is enriched in several tumor types including CRC and correlates with suppression of the anti-tumor immune response. In the large intestine, thymic Tregs (tTregs Helios^+^) and peripheral Tregs (pTregs Helios^−^) coexist and maintain intestinal homeostasis under steady state conditions. The recruitment of Treg cells to the intestine is orchestrated by the CCR9/CCL25 axis, which is potentiated under inflammatory conditions. Interestingly, the balance between cytotoxic CD8^+^ T cells and Tregs within the tumor microenvironment is critical for antitumor immunity and cancer progression. An elevated CD8^+^/Treg ratio has been associated with improved clinical outcomes in various cancers, including CRC. Therefore, here we investigate the potential role of chemokine receptor CCR9 on CD8^+^/Treg ratio and the effect of the recruitment of Treg subpopulations (Helios^+^ and Helios^−^) into the tumor microenvironment using the AOM/DSS induced colitis-associated colorectal cancer murine model. Our findings reveal that CCR9 deficiency leads to distinct alterations in the CRC microenvironment, characterized by decreased intratumoral Tregs Helios^+^. Also, the lack of the receptor leads to an improvement of the antitumor immune response, increasing the CD8^+^/Treg ratio within the tumor immune infiltrate. These results underscore the importance of CCR9 in shaping the immune microenvironment during CRC development.

## Introduction

Colorectal cancer (CRC) is the leading cause of cancer-related deaths among men and women in developing countries [1]. Several risk factors, such as diet, genetic predisposition and chronic inflammation have been strongly linked to CRC development [2–4]. It is well documented that chronic colonic inflammation due to ulcerative colitis or Crohn’s disease increases the risk of developing colorectal cancer. The relationship between inflammation and cancer is multifaceted, involving various types of inflammatory cells and molecules. Inflammatory cells play a crucial role in shaping the tumor microenvironment, through the secretion of growth factors, angiogenic mediators, chemokines, cytokines, etc., that contribute to the survival, growth and enhanced invasiveness of tumor cells [5].

The evolution of cancer is highly dependent on the tumor microenvironment, where certain tumor-infiltrating immune cells hold prognostic value. Among these, CD8^+^ T-cells and regulatory T cells (Tregs) play pivotal roles in either promoting anti-tumor immunity or facilitating immune escape, respectively, making them crucial for understanding the immune microenvironment within the tumor [6,7]. CD8^+^ T-cells are the main cells responsible for the antitumor response. These cells recognize antigens presented by MHC-I on the surface of tumor cells and, upon recognition, induce apoptosis in tumor cells through the release of perforins and granzymes. Additionally, CD8^+^ T-cells produce cytokines such as IFN-γ and TNF-α, which enhance antitumor activity by inhibiting tumor cell proliferation and increasing the expression of MHC-I on tumor cells. The presence of CD8^+^ T-cells is associated with improved prognosis in many types of cancer, including CRC [8]. On the other hand, Tregs are a subpopulation of CD4+ T cells characterized by the expression of the transcription factor FoxP3, which is essential for their development and function [9]. These cells play multiple roles in modulating immune responses, mainly suppressing the activation, function, and proliferation of other immune cells, including T cells, B cells, and antigen-presenting cells. Among their main functions are the suppression of autoimmune responses, the maintenance of tolerance to self and exogenous antigens and the regulation of inflammatory responses [10].

The intestinal tract constitutes a specially tolerogenic milieu, involving several mechanisms that maintain tolerance towards both microbiota-derived and dietary antigens, under homeostatic conditions [11]. In the large bowel, two subpopulations of Tregs have been identified: thymic Tregs (tTreg CD4^+^ FoxP3^+^ Helios^+^) that arise in the thymus and recognize preferentially self-antigens, and peripheral Treg (pTregs CD4^+^ FoxP3^+^ Helios^−^) that are generated in the periphery from naïve T cells and recognize antigens from diet and commensal microbiota promoted by the intestinal milieu, which is highly enriched in TGF-β and Retinoic acid [11–13].

The role of Treg cells has been widely studied in cancer progression, as they emerge as key regulators of antitumor immunity. Indeed, these cells are highly enriched within the microenvironment of growing tumors, and their presence correlates with a poor prognosis in many cancer types [7,14–16]. During CRC development, CD8^+^ T cells and highly suppressive Tregs (mostly tTregs) are recruited within the tumor microenvironment [17,18]. However, the specific role of Treg cells in CRC remains controversial. In this context, it has been established that the CD8^+^/Treg cell ratio, may serve as a quantitative measure of the balance between anti-tumor and pro-tumor immune activity: a higher CD8^+^/Treg cell ratio indicates an immune microenvironment that favors the tumor clearance while a lower ratio indicates a predominance of immune suppression which promotes tumor progression [19,20].

The recruitment of immune cells both under homeostatic or pathological conditions is finely orchestrated by several cell trafficking mechanisms, including homing molecules like integrins and chemokines [21–24]. The chemokine receptor CCR9 in the gastrointestinal tract is primarily expressed in the small intestine, where it plays a pivotal role in recruiting various immune cells by interacting with its ligand CCL25. However, in the colon, CCR9 and its ligand are expressed during inflammatory processes, actively participating in the recruitment of diverse immune cells [25–27]. Indeed, Wurbel et al. showed that although under homeostatic conditions CCR9 expression was not observed in the colon, it was induced in a DSS colitis model, particularly in plasmacytoid dendritic cells and intraepithelial T lymphocytes [28].

Studies in patients with CRC describe that tumor-infiltrating immune cells exhibit a distinct pattern of chemokine receptors compared to healthy tissue. Among these receptors, it has been identified that the chemokine receptor CCR9 is expressed differentially in the tumor and non-tumor areas, showing a decreased percentage of CCR9^+^ CD8^+^ T cells in CRC compared to normal tissue [29]. However, it is unknown the potential role of CCR9 on Treg recruitment during CRC development.

This study investigates the potential role of CCR9 in the recruitment of immune cells known to be crucial during CRC progression (CD8^+^ T cells versus Tregs), using the Colitis-Associated Colorectal Cancer mice model. Our results reveal that CCR9 controls the immune microenvironment of colorectal cancer differentially affecting both CD8^+^ T cells and Tregs, and that its absence results in an increase in the CD8^+^/Treg cell ratio, which promotes an enhanced anti-tumoral response.

## Materials and methods

### Mice

The C57BL/6 CCR9-deficient mice were generated and kindly provided by Wurbel et al. through the deletion of exon 3 of the *CCR9* gene, which encodes 362 of the 369 amino acids, using homologous recombination. The deletion was confirmed by PCR and Southern blot analysis. The *CCR9*-deficient mice are viable, fertile, and exhibit no morphological alterations or developmental abnormalities [30]. To carry out the CRC model in the Balb/c strain, C57BL/6 CCR9-deficient mice were backcrossed with Balb/c mice and animals from the F10 generation were used for experiments. CCR9^+/−^ and CCR9^−/−^ mice were housed and bred under specific pathogen-free conditions in the animal facility unit of Instituto de Investigaciones Biomédicas, Universidad Nacional Autónoma de México, (UNAM). This study was performed in strict accordance with the recommendations in the Guide for the Care and Use of Laboratory Animals of the Biomedical Research Institute (protocol ID 124).

### Experimental AOM/DSS induced colitis-associated colorectal cancer murine model

Experiments were conducted using mice aged between six to ten weeks, weighing up to 22 grams. The animals were divided into three experimental groups: Control group (CTRL), Colitis group (DSS), and Colitis-Associated Colorectal Cancer group (AOM/DSS). The Colitis-Associated Colorectal Cancer model was initiated with a single intraperitoneal of 10 mg/kg of azoxymethane (Sigma-Aldrich), one week later, DSS treatment was initiated by providing 2% DSS (36–40 kDa, Affymetrix) in drinking water for 7 days, followed by a 7-day period with normal water. This cycle was repeated three times. After the third cycle, mice were given normal water for the remaining period to the endpoint. The study duration was 60 days, during which tumors formed in the large intestine of 100% of the animals without dramatic weight loss or signs of pain or suffering. Body weight was assessed three times per week, and the final analysis was performed on day 60. Throughout the study, the health and behavior of the animals were monitored daily by the animal facility staff to minimize suffering. To alleviate suffering, the following humane endpoints were established: a marked decrease in activity, aggressiveness, changes in grooming behavior, reduced body temperature, abnormal posture, and a drastic weight loss exceeding 15% of body weight. If any animal met the criteria for a humane endpoint, euthanasia was performed within 30 minutes [31].

If a study animal died before meeting the euthanasia criteria based on the humane endpoints, the event was documented, and the signs and symptoms observed prior to death were investigated. A necropsy was then performed to determine the cause of death and rule out unexpected or external factors, such as infections or failures in the experimental protocol. All the necessary adjustments to the protocol were made during the standardization phase, and the humane endpoints were also revised. The experimental protocol determined a sample size of six animals per treatment group. All animals were euthanized at the end of the study using a carbon dioxide (CO_2_) chamber, following a gradual fill rate to minimize distress and confirmatory checks to ensure death. The use of anesthesia or analgesia was not necessary during the euthanasia process, as CO₂ exposure is an approved method that does not require prior anesthesia. No animal died due to external or unexpected causes, and the survival rate at the end of the experimental protocol was 100%. The animals were housed in a controlled environment, with environmental enrichment provided, including toys and nesting materials to stimulate natural behaviors such as exploration, exercise, and social interaction, following institutional guidelines for housing conditions. All personnel involved in developing experimental protocols at the animal facility were rigorously trained in animal care and handling by the institution’s animal care staff. All findings were reported to the Institutional Animal Care and Use Committee (CICUAL).

### Preparation of cell suspensions

Single-cell suspensions from colonic lamina propria were prepared according to the method previously described. Initially, large intestines were flushed and opened longitudinally to remove intraepithelial lymphocytes and epithelial cells from the lamina propria. Intestinal pieces were incubated with shaking at 150 rpm at 37°C for 30 minutes in calcium and magnesium free Hank’s balanced salt solution supplemented with 5% FBS, 5 mM EDTA (Sigma-Aldrich), 10mM HEPES, and 2 mM DTT [32,33]. The suspension was discarded, and the remaining tissue was collected. The distal portion of the large intestine was dissected and fragmented into 1–2 mm pieces. Also, the distal fragments from AOM-treated mice were dissected into tumor and peri-tumoral region. Tissues fragments were collagenase-digested with RPMI 1640 containing 10% FBS and 37 U/mL collagenase type IV from Clostridium (Sigma-Aldrich) for 35 minutes at 37°C with shaking at 400 rpm. Cells in suspension were collected by passage through a 100 μm cell strainer (BD Biosciences Ltd, Oxford, UK). The cell suspension was washed and resuspended in complete media containing 10% FCS and antibiotics (penicillin 50 U/ml, streptomycin 50 U/ml) and stored at 4°C until staining for flow cytometry [34].

### Histological analysis and inflammatory score

Distal colon samples were dissected and washed with PBS, fixed in 10% formalin, and cryopreserved in an embedding medium for frozen tissues (Tissue-Plus; Fisher HealthCare). Cryostat sections of 4 mm were obtained and fixed for H&E staining. Inflammatory and hyperplasia scoring was conducted by a pathologist, blinded to the conditions, as follows: Inflammation was defined by the presence of infiltrating cells; 0 for no infiltrate; 1 for low infiltrate limited to submucosa; 2 for significant inflammatory cells in submucosa limited to focal areas; 3 for infiltrate present in submucosa and colonic lamina propria; 4 for a large amount of infiltrate in submucosa, lamina propria, and surrounding areas. Crypts damage was defined as follows: 0 for none; 1 for minimal crypt damage; 2 for larger spaces between crypts, loss of goblet cells, some shortening of crypts; 3 for large areas without crypts, surrounded by normal crypts; 4 for no crypts. Ulceration score was defined as follows: 0 for none; 1 for some small, focal ulcers; 2 for frequent small ulcers; 3 for large areas lacking surface epithelium. Hyperplasia and goblet cells loss were defined as follows: 0 for normal crypts; 1 for elongated crypts and normal goblet cells present; 2 for multifocal areas with elongated crypts and normal goblet cells present; 3 for multifocal elongated crypts and reduced goblet cells present; 4 for extensive areas with elongated crypts, crypts fusion, loss of structure and absent goblet cells [35,36]. Colonic mucosa adenocarcinomas were confirmed according to Boivin et al. criteria [37].

### Flow cytometry analysis

Cell suspensions from lamina propria, were stained with Zombie Aqua fixable dye and anti-CD8 PECy7 from Biolegend; anti-CD4 APC-Cy7 from Tonbo Biosciences and anti-CD25 PE-Cy5.5 from Thermo Fisher Scientific for 20 min at 4°C; For intracellular staining, cells were fixed and permeabilized (CAT Fix/Perm permeabilization kit, Tonbo Biosciences) and stained with anti-FoxP3 APC from Tonbo Biosciences and anti-Helios FITC from Biolegend 1h at room temperature and posterior intracellular staining for 30 min at 4°. Samples were run on an Attune Next (Thermo Fisher Scientific). Samples were acquired in an Attune Next Acoustic Focusing Flow Cytometer (Thermo Fisher Scientific) and analyzed using Flowjo 10.0 software (BD Biosciences).

### Statistical analysis

In vivo experiments were conducted twice. Data are presented as mean ± SD. Two-way ANOVA analysis of variance, followed by Sidak post hoc test, was used to analyze body weight loss data. The Mann-Whitney test was used to compare differences between two groups (CCR9^+/−^ vs. CCR9^−/−^). A p value < 0.05 was considered significant. All statistical analyses were performed using GraphPad Prism version 8.0 for Windows (GraphPad software, San Diego, CA, USA).

## Results

### CRC development by AOM/DSS is favored by CCR9 expression

**Fig 1:**
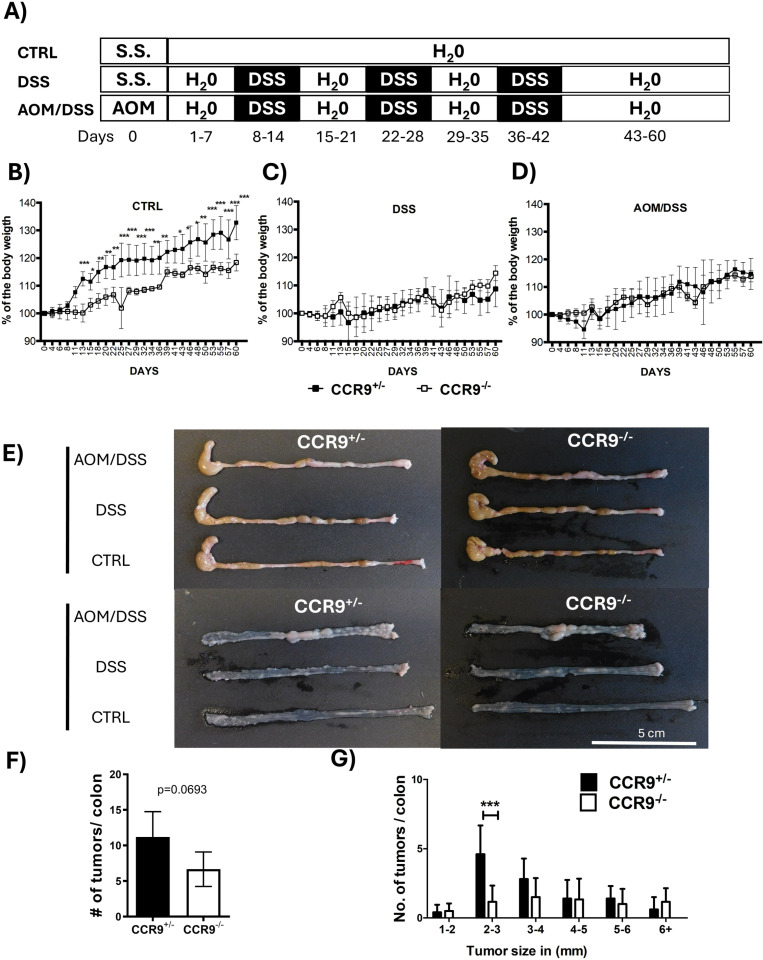
CCR9 expression favors the development of CRC. Schematic representation of CRC induction protocol in Balb/c CCR9^+/−^ and CCR9^−/−^ mice, (A). Changes in body weight in control groups (B), DSS treated groups (C) and AOM/DSS treated groups (D). Representative images of large bowels harvested in CCR9^+/−^ and CCR9^−/−^ at day 60 after DSS or AOM/DSS treatment (E). Number of total tumors (E), and number of tumors by size in mm (F) after AOM/DSS treatment. Representative data shown from 2 independent experiments, n = 5–6 animals per group. Data are represented as mean ± SD. *p < 0.05, **p < 0.01, ***p < 0.001.

To understand the role of CCR9 in the CRC development, we first established the colitis-associated colorectal cancer model, using CCR9^+/−^ and CCR9^−/−^ mice administering AOM/DSS (Fig 1A). In the absence of CCR9, control mice experienced a significantly reduced weight gain compared to CCR9^+/-^ mice (Fig 1B). Both strains were susceptible to DSS and AOM/DSS as evidenced by body weight loss (Fig 1C, D).

To evaluate the effect of CCR9 on tumor growth, we isolated colon tissue from mice at the end of the experimental model (Day 60). Interestingly, although 100% of both CCR9^+/−^ and CCR9^−/−^ mice developed tumors after AOM/DSS treatment at the distal region of the colon, as expected (Fig 1E) [38]. CCR9^−/−^ mice showed a trend to develop less tumors than CCR9^+/−^ mice (p=0.0693) (Fig 1E, F) and developed significantly less tumors of 2–3 mm in size (Fig 1F), indicating that expression CCR9 may favor colon cancer development.

### Inflammatory damage is independent of CCR9 expression during CRC development in Balb/c mice

**Fig 2:**
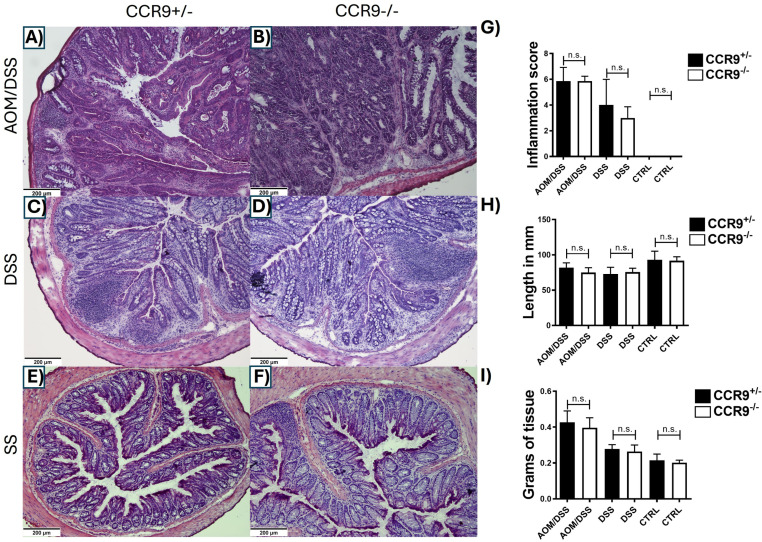
Lack of CCR9 does not affect the inflammatory features in the colon after DSS or AOM/DSS treatment. Representative H&E staining of colonic tissue from CCR9^+/−^ (A, C, E) and CCR9^−/−^ mice (B, D, F) from control, DSS and AOM/DSS treatments (10X-magnification). Inflammatory score (G), colon lengths (in mm) (E), colon weight (in grams), from CCR9^+/−^ and CCR9^−/−^ mice after treatments (F). Representative data shown from 2 independent experiments, n = 5–6 animals per group. Data are represented as mean ± SD. *p < 0.05, **p < 0.01, ***p < 0.001.

In this CRC model, inflammation plays an important role in the regulation of tumor development. Therefore, to examine the impact of CCR9 in this process, we assessed the inflammatory histological characteristics in the colon. Histopathological features during colon inflammation involve immune cell infiltration, epithelial hyperplasia with goblet cells loss, crypts damage, and ulcer formation [35,36]. Thus, we analyzed these histologic parameters in H&E sections (Fig 2A–F). The inflammatory score after DSS or AOM/DSS treatment were similar in both CCR9^+/−^ and CCR9^−/−^ mice, accompanied with the presence of tumors in the AOM/DSS groups (Fig 1G), our results suggest that CCR9 expression is dispensable for the features of inflammatory damage in this model. Also, colon shortening is a well-documented macroscopic sign of inflammation [35,39,40]. We observed a significant colon shortening in groups under DSS or AOM/DSS treatment, with no significant differences between CCR9^+/−^ and CCR9^−/−^ mice (Fig 2H).

Another feature of colonic inflammation is the increased weight in colonic tissue due to the recruitment of inflammatory cells and fluids, this weight gain is markedly higher in the AOM/DSS groups due to the presence of tumors. However, no discernible differences were observed when CCR9^+/−^ and CCR9^−/−^ groups are compared (Fig 2I).

### CCR9 deficiency increases the percentage of CD8^+^ effector T cells within the CRC tumors

**Fig 3:**
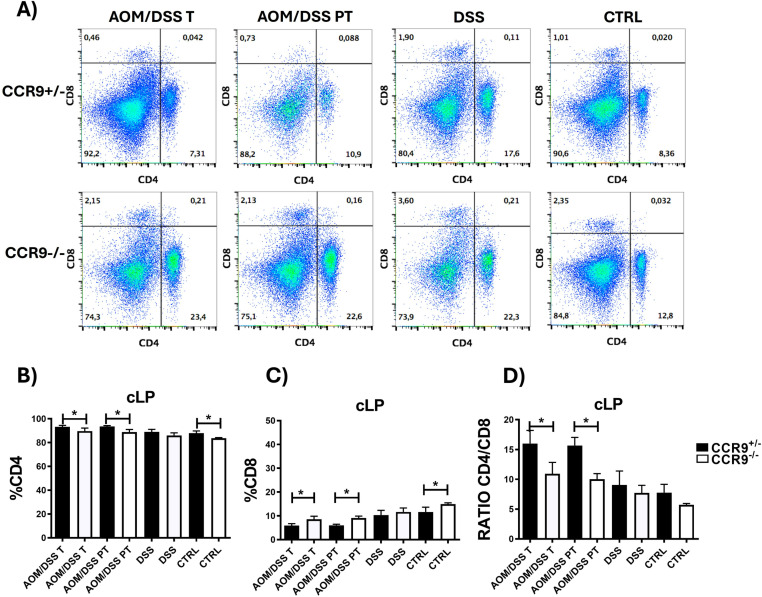
Loss of CCR9 increases CD8 ^**+**^ **effector T cells within colorectal cancer tumors (CRC).** Representative plots of CD4^+^ and CD8^+^ T cells (A), Frequencies of CD4^+^ cells (B), CD8^+^ cells (C) and CD4/CD8 ratio (D) in cLP, tumoral (T) and peri tumoral (PT) regions after treatments in CCR9^+/−^ and CCR9^−/−^ mice. CD4^+^ and CD8^+^ frequencies were calculated from total T cell events. Representative data shown from 2 independent experiments, n = 3–6 animals per group. Data are represented as mean ± SD.*p < 0.05.

Although no apparent differences in the inflammatory response were observed when comparing CRC tumors from CCR9^+/−^ or CCR9^−/−^ mice, it has been described that CD4^+^ and CD8^+^ T cells recruited to the colonic mucosa during CRC changes their chemokine receptor profile compared to unaffected mucosa. Interestingly, the authors showed a decrease in the percentage CCR9^+^ CD8^+^ cytotoxic T lymphocytes, although the functional relevance of this subpopulation was not evaluated [29]. To determine whether CCR9 is involved in the recruitment of CD4^+^ and CD8^+^ effector T cells during CRC development, we analyzed the cLP infiltrate under basal (CTRL), inflammatory (DSS) and cancer promoting conditions (AOM/DSS), using flow cytometry (Fig 3A). From the AOM/DSS treated cLP tissue mice, we isolated the tumor zone (T) and the peri-tumoral zone (PT). We found that in the absence of CCR9, there is a significant increase of CD8^+^ T cells recruited to both the tumor and peri-tumoral zone zones and therefore, with a concomitant decrease in the frequencies of CD4^+^ T cells, as expected. Interestingly, the frequencies of CD8^+^ vs CD4^+^ T cells were similarly affected in the cLP of control but not DSS treated mice.

These data suggest that CCR9 is involved in the migration of CD4^+^ and CD8^+^ effector T cells during CRC progression but does not appear to depend only on the inflammatory process within the cLP (Fig 3B,C). Remarkably, the CD4/CD8 ratio, which indicates the immune state [41], is significantly lower in the tumor and peri-tumoral zone of CCR9^−/−^, suggesting that CCR9 expression controls the immune balance in colon cancer, and showing an enrichment of CD8^+^ T cells when CCR9 is absent (Fig 3D).

### CCR9 controls the recruitment of Tregs subpopulations (Helios^+^ and Helios^−^) during CRC

Our data showed the relevance of CD8^+^ T cell recruitment within the tumor microenvironment, however accumulating evidence has demonstrated the role of other immune cells in the balance between an efficient anti-tumoral response and cancer progression. Specifically, the increase of intratumoral CCR8^+^ Tregs has been associated with a poor prognosis in various cancers including CRC [42].

**Fig 4:**
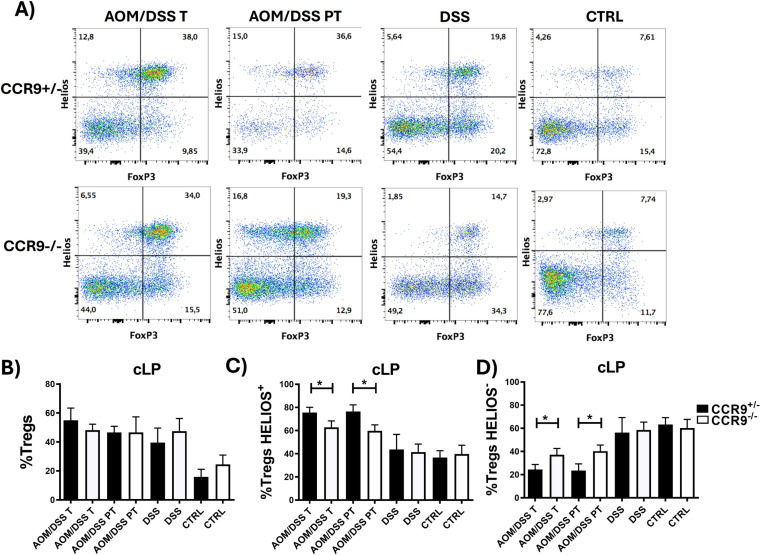
CCR9 controls the recruitment of Treg subpopulations during CRC development. Representative plots showing Treg FoxP3^+^ Helios^+^ and Helios^-^ subpopulations (gated from CD4^+^ cells) (A). Frequencies of Treg FoxP3^+^ cells (B), Treg FoxP3^+^ Helios^+^ cells (C) Treg FoxP3^+^ Helios^-^ cells (D) in cLP, tumoral (T) and peri tumoral (PT) regions after treatments in CCR9^+/−^ and CCR9^−/−^ mice. Representative data shown from 2 independent experiments, n = 3–6 animals per group. Data are represented as mean ± SD. *p < 0.05.

Tregs in colonic epithelia can be subdivided in two principal groups: Helios^+^ Tregs, also named tTreg (CD4^+^ FoxP3^+^ Helios^+^), and Helios^−^ Tregs, also named pTregs (CD4^+^ FoxP3^+^ Helios^−^) [11,12]. Under homeostatic conditions pTregs comprise the majority of Tregs, while during CRC development tTregs increase dramatically [18]. However, the mechanisms that control these changes are not yet well understood. Therefore, we next investigated whether CCR9 may regulate the balance between tTregs and pTregs in the cLP during CRC development using flow cytometry. As shown in Fig 4A, the total frequency of Tregs (CD4^+^FoxP3^+^) in cLP of mice treated with DSS or AOM/DSS was significantly increased after DSS or AOM/DSS treatment, as previously described [43–45]. Moreover, we did not find significant differences in Treg recruitment between CCR9^+/−^ and CCR9^−/−^ mice (Fig 4 B).

As mentioned above, different Treg subsets are recruited in the colon mucosa under homeostatic or inflammatory/CRC conditions, therefore, we analyzed the proportion of Helios^+^ and Helios^-^ Treg subpopulations during CRC development and investigated whether CCR9 could participate in this process. Our results show that the absence of CCR9 leads to a decrease of Helios^+^ Tregs (tTregs) in both intra-tumoral and peri-tumoral region, while in parallel the frequencies of Helios^-^ Tregs (pTregs) were significantly increased (Fig 4C,D).

It has been reported that Tregs Helios^+^ express high levels of CD25, which allows them to respond to IL-2 signals in their microenvironment. CD25 expression is critical for the development and maintenance of Tregs, as its expression correlates with the suppressive activity. Therefore, we examined whether the lack of CCR9 was involved in the expression of CD25 in Treg Helios^+^ and Helios^−^ subpopulations. As expected, most Tregs Helios^+^ express higher CD25 compared to Tregs Helios^−^, although we did not find significant differences between CCR9^+/−^ and CCR9^−/−^ mice, indicating that CCR9 does not affect CD25 expression (S1 Fig).

In summary, CCR9 plays an important role in the balance of Tregs Helios^+^ and Helios^−^ in cLP during CRC development, decreasing the frequencies of Tregs Helios^+^ that have been previously described to be highly suppressive in CRC [18].

### The absence of CCR9 results in an increased intratumoral CD8^+^/Treg Ratio

It is well documented that the CD8^+^/Treg ratio indicates the balance between the anti and pro-tumoral immune response, which reflects the process called “cancer immune surveillance”. Thus, a higher CD8^+^/Treg ratio is associated with a more favorable prognosis, suggesting an enhanced anti-tumor immune response [46].

**Fig 5:**
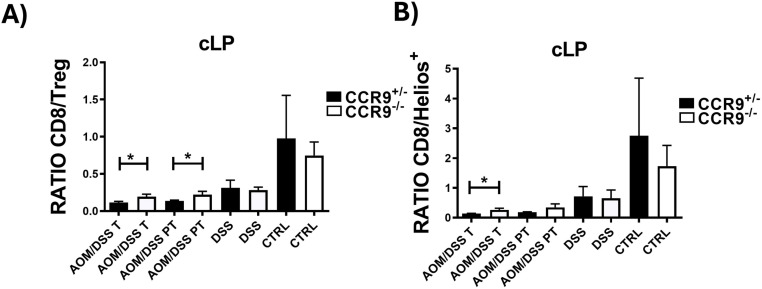
The absence of CCR9 results in an increased intratumoral CD8 ^**+**^**/Treg Ratio.** **CD8**^**+**^**/Treg Ratio** (A) and CD8^+^/Treg Helios^+^ Ratio (B) in cLP, tumoral (T) and peri tumoral (PT) regions after treatments in CCR9^+/−^ and CCR9^−/−^ mice. Representative data shown from 2 independent experiments, n = 3–6 animals per group. Data are represented as mean ± SD. *p < 0.05.

Our results demonstrate that the absence of CCR9 significantly increases the CD8^+^/Treg ratio in the tumor and peritumoral space, indicating that CCR9 plays a fundamental role in modulating the anti-tumoral immune response in CRC (Fig 5A). Moreover, as the suppressive Helios^+^ Treg are the main subpopulation increased in CRC, we next evaluated the CD8^+^/ Treg Helios^+^ ratio in this model. Interestingly, we found that this ratio is significantly increased in the intratumoral space of CCR9^−/−^ mice compared to CCR9^+/-^ mice, indicating that the recruitment of Tregs Helios^+^ into the intratumoral microenvironment is modulated by CCR9 (Fig 5B). These results suggest that CCR9 controls the antitumoral immune response through the modulation of the balance between Treg cell populations and CD8^+^ effector T cells.

## Discussion

Colon cancer is the second cause of death related to cancer, and its incidence is rising, due to lifestyle and dietary changes, which results in microbiota alterations, dysbiosis, and a concomitant loss of colonic homeostasis [1]. Indeed, increased intestinal inflammation has been considered as a susceptibility factor in CRC development [47]. CCR9 is a key regulator of colon inflammation, contributing to the pathogenesis of inflammatory bowel disease (IBD) through the recruitment of T cells into the colon mucosa. Additionally, the expression of the CCR9 ligand, CCL25, correlates with the severity of the disease [48–50]. On the other hand, Wurbel et al. reported an increased large bowel inflammation in the CCR9^−/−^ B6 mice after DSS-induced chronic and acute colitis compared to wild-type mice [28,51]. In contrast, in our study, CCR9^−/−^ Balb/c mice did not show significant differences in inflammatory signs after DSS treatment (Fig 2). This could be attributable to differences in genetic background and susceptibility to DSS treatment between C57BL/6 and Balb/c mice, as well as to the different time of subpopulation analysis [52–54].

Although control CCR9^−/−^ Balb/c mice showed a delay in weight gain, this might be related to intrinsic defects in the intestine-associated lymphoid tissue in the small intestine. Previous studies have reported that CCR9^−/−^ displayed a reduced number of antibody-secreting plasma cells and fail to mount a regular IgA response to oral antigens [55]. This failure may affect the maintenance of intestinal health, the induction of immune responses, and the tolerance to gut microbiota and food antigens that could alter the nutritional state of the mice.

Several studies have reported the role of CCR9 in controlling inflammation in the colon. Wurbel et al. showed that the CCL25/CCR9 interaction is dispensable for the function of tTregs and the induction of pTregs in the colonic lamina propria (cLP) during chronic colitis induced by the transfer of CD45RB CD4^+^ T cells into either Rag1^−/−^ or Rag1^−/−^ CCL25^−/−^ recipient mice [51]. Our results with DSS-treated mice are consistent with this report, as we did not find any differences between CCR9^+/−^ and CCR9^−/−^ mice.

Nevertheless, we need to consider that chronic inflammation and cancer are two distinct yet interrelated biological processes. In the AOM/DSS model, inflammation promotes cancer development [38,47]. Once cancer is established, tumors create an immunosuppressive environment through the secretion of cytokines like TGF-β and IL-10, and the recruitment of Tregs [56]. Here, we demonstrate that in the absence of CCR9 during CRC, there is an increased proportion of Helios^−^ Tregs (presumably pTregs) and a concomitant decrease in the frequencies of Helios^+^ Tregs (tTregs). The increased pTregs observed in CCR9^−/−^ mice align with reports showing that the CCR9/CCL25 axis has a negative effect on pTreg development [57].

There is growing evidence showing that cancer progression is highly associated with the immune infiltrate recruited to the tumor, which is regulated by chemokines and their receptors [47]. In cLP the recruitment of CD8^+^ T cells and Tregs is orchestrated by different chemokine receptors that vary under homeostatic or pathological conditions, such as in cancer [58]. In CRC, it has been reported that several chemokine receptors regulate Treg (CCR8, CCR6, CCR4, CCR5 and CXCR4) and CD8^+^ T cell (CXCR5, CCR2 and CCR9) migration [29,42,59–62]. Although CCR9 has been mostly related to immune subpopulations recruited to the small intestine, both under homeostatic and inflammatory conditions [25], we cannot exclude the possibility that CCR9 could be involved in CRC, as this cancer is highly associated with inflammation, and CCR9^−/−^ mice develop a severe colitis phenotype, potentially predisposing them to CRC development. Indeed, previous studies using CCR9^−/−^ mice showed that IL-10 producing cells might control DSS-induced inflammation in the colon, suggesting that CCR9^+^ immune regulatory cells, including Tregs, could play a role in maintaining intestinal homeostasis [28,51,57].

Although some studies have used immunohistochemical analysis, which offers spatial information of the cell-cell interactions occurring within the tumor microenvironment, localization of immune subpopulations is not a static process and may change during CRC progression. Furthermore, it encounters certain technical limitations in comparison to flow cytometry, particularly in the simultaneous analysis of multiple markers such as CD25, Foxp3, Helios, and CCRs. 

This is specially relevant since the tumor microenvironment is characterized by hypoxia, cytokine-mediated inflammation, and a unique metabolic milieu that modifies Treg function and induces a unique chemokine receptor profile [47,63,64]. It has been reported that Treg frequencies increase within CRC tumors when compared to unaffected tissues, and that intratumoral Tregs display a highly immune-suppressive phenotype (Helios^+^, PD-1^high^, CTLA-4^high^) compared to non-tumoral tissue Tregs [18]. In this context, intratumoral Tregs expressing CCR8 exhibit a profoundly suppressive phenotype characterized by PD-1 dependency [42].Moreover, inhibition of CCR8 in Tregs results in an enhanced anti-tumoral immune response [65]. Given the role of CCR9 in controlling inflammatory processes in the colon, we hypothesized that CCR9 could also participate in the recruitment of the Treg subpopulations that modulate the development of CRC, Our findings demonstrate that the absence of CCR9 promotes the accumulation of Helios^-^ Tregs and a decreased proportion of highly suppressive Helios^+^ Tregs within the tumor microenvironment. This alteration may contribute to a less immunosuppressive milieu and may potentially facilitate anti-tumor immunity. This could be further proven by performing a functional evaluation of these subpopulations using isolated Tregs from Foxp3 and Helios reporter mice that could be used in *in vitro* suppression assays.

In human CRC patients, tumoral tissue shows a significant decrease in the percentage of CCR9^+^ CD8^+^ T lymphocytes compared to non-tumoral tissue, suggesting that they may be biologically relevant for the anti-tumoral immune response [29]. The authors proposed a novel immune evasion mechanism used by tumor cells, where the manipulation ofchemokine/chemokine receptor axis seems to represent a limiting mechanism for anti-tumoral immune response, as increased expression of CCL25 (CCR9 ligand) surrounding CRC epithelium and downregulation of intratumoral CCL25 prevent the entry of cytotoxic CCR9 CD8^+^ T cells into the tumor [29].

It is widely accepted that the anti-tumoral response is primarily associated with infiltrating CD8^+^ effector T cells, while the pro-tumoral response is strongly promoted by the presence of Treg cells. Indeed, it has been shown that the depletion of Treg cells within the tumor improves the response by CD8^+^ T cells [45]. Therefore, in many types of cancers the CD8^+^/Treg ratio has been widely used as an immune score that represents the tumor microenvironment and that has proven to be very efficient in-patient prognosis and to predict the response to neoadjuvant chemotherapy [19,20]. In our study, the lack of CCR9^−/−^ led to an increased CD8^+^/Treg cell ratio, indicating a stronger anti-tumoral immune response, which explains the reduced tumor number observed in these mice.

Our current findings contribute to the understanding of the complex immune microenvironment that takes place in CRC. However, we cannot rule out that the effects observed in the Tregs of CCR9^−/−^ mice during CRC development may involve both CCR9-direct and indirect mechanisms. Previous reports have indicated that the absence of CCR9 alters the recruitment of innate immune cells such as plasmacytoid dendritic cells (pDCs), neutrophils, conventional dendritic cells (cDCs), and macrophages [51]. All these cells have been reported in colon cancer and can affect Treg cell dynamics; for example, pDCs and cDCs can promote the induction and expansion of Tregs; macrophages, depending on their polarization state (M2 or M1), can either enhance or suppress Treg activity, respectively, and neutrophils, can recruit Tregs into tumors through CCL17 [66–68].

Our data demonstrate that CCR9 shapes the tumor microenvironment, affecting the recruitment of effector CD4^+^, CD8^+^ and Treg cells subsets. This was demonstrated by our results in the AOM/DSS model, where in CCR9^−/−^ mice displayed a reduced number of tumors, which was associated with an increase in the intratumoral CD8^+^/Treg ratio, indicative of an enhanced immune response against tumors. However, it is important to recognize that other chemokine receptors may also play a role in this process. For example, it has been reported that chemokine receptors CCR2, CCR5, and CXCR5 modulate the recruitment of CD8^+^ T cells, while CCR6 and CXCR4 are important for Treg recruitment in CRC [29,59,62]. 

Understanding the contribution of chemokine receptors, such as CCR9, in the immune response to tumors is crucial for developing therapeutic strategies to improve the patient’s outcomes. Furthermore, the role of CCR9 as a potential therapeutic target in intestinal inflammation has also been established. Although pharmacological inhibitors of this receptor have been tested, their efficacy has fallen short of expectations, as heterogeneous results have been reported in clinical trials [48,49]. Consequently, novel strategies to modulate CCR9 activity are required. It is essential to determine whether the use of CCR9 antagonists, in combination with established therapies, could decrease the recruitment of Treg cells to tumor sites while increasing CD8^+^ T cell infiltration. Moreover, the effectiveness of these inhibitors remains unexplored in the context of CRC, representing a promising approach for enhancing the anti tumor response. However, further studies are required to validate this hypothesis and to determine whether CCR9 expression could serve as a predictive marker for the response to neoadjuvant chemotherapy, with potential prognostic value in CRC patients.

## Supporting Information

S1 Fig Helios-Positive Treg Cells express high levels of CD25 in a CCR9 independent way. Representative plots showing CD25 expression in Tregs FoxP3^+^ Helios^+^ and Helios^−^ cells (A,B), frequencies of Tregs FoxP3^+^ Helios^+^ CD25^+^ cells (C), frequencies of Tregs FoxP3^+^ Helios^−^ CD25^+^ cells (D) in cLP, tumoral (T) and peri tumoral (PT) regions after treatments in CCR9^+/−^ and CCR9^−/−^ mice. Representative data shown from 2 independent experiments, n = 3–6 animals per group. Data are represented as mean ± SD.*p < 0.05. (TIF)

## References

[1] Sung H, Ferlay J, Siegel RL, Laversanne M, Soerjomataram I, Jemal A, et al. Global cancer statistics 2020: GLOBOCAN estimates of incidence and mortality worldwide for 36 cancers in 185 countries. CA Cancer J Clin. 2021;71(3):209–49. doi: 10.3322/caac.21660 33538338

[2] Lasry A, Zinger A, Ben-Neriah Y. Inflammatory networks underlying colorectal cancer. Nat Immunol. 2016;17(3):230–40. 26882261 10.1038/ni.3384

[3] Shah SC, Itzkowitz SH. Colorectal cancer in inflammatory bowel disease: mechanisms and management. Gastroenterology. 2022;162(3):715–730.e3. 34757143 10.1053/j.gastro.2021.10.035 PMC9003896

[4] Vernia F, Longo S, Stefanelli G, Viscido A, Latella G. Dietary factors modulating colorectal carcinogenesis. Nutrients. 2021;13(1):1–10. doi: 10.3390/nu13010001 PMC7824178 33401525

[5] Mantovani A, Allavena P, Sica A, Balkwill F. Cancer-related inflammation. Nature. 2008;454(7203):436–44. doi: 10.1038/nature07205 18650914

[6] St Paul M, Ohashi PS. The roles of CD8+ T cell subsets in antitumor immunity. Trends Cell Biol. 2020;30(9):695–704. doi: 10.1016/j.tcb.2020.06.003 32624246

[7] Li C, Jiang P, Wei S, Xu X, Wang J. Regulatory T cells in tumor microenvironment: new mechanisms, potential therapeutic strategies and future prospects. Mol Cancer. 2020;19(1):116. doi: 10.1186/s12943-020-01234-1 32680511 PMC7367382

[8] Angell HK, Kirkwood K, Meyerhardt JA, Norton L, Sargent DJ, Schmiegelow K, et al. The immunoscore: colon cancer and beyond. Clin Cancer Res. 2020;26(2):332–9. doi: 10.1158/1078-0432.CCR-19-1740 31413009

[9] Fontenot JD, Gavin MA, Rudensky AY. Foxp3 programs the development and function of CD4^+^ CD25^+^ regulatory T cells. Nat Immunol. 2003;4(4):330–6. doi: 10.1038/ni904 12612578

[10] Sakaguchi S, Yamaguchi T, Nomura T, Ono M. Regulatory T cells and immune tolerance. Cell. 2008;133(5):775–87. doi: 10.1016/j.cell.2008.05.009 18510923

[11] Traxinger BR, Richert-Spuhler LE, Lund JM. Mucosal tissue regulatory T cells are integral in balancing immunity and tolerance at portals of antigen entry. Mucosal Immunol. 2022;15(3):398–407. doi: 10.1038/s41385-021-00471-x 34845322 PMC8628059

[12] Thornton AM, et al. Expression of Helios, an Ikaros transcription factor family member, differentiates thymic-derived from peripherally induced Foxp3 T regulatory cells. J Immunol. 2010;184(7):3433–41. 20181882 10.4049/jimmunol.0904028 PMC3725574

[13] Whibley N, Tucci A, Powrie F. Regulatory T cell adaptation in the intestine and skin. Nat Immunol. 2019;20(4):386–96. 30890797 10.1038/s41590-019-0351-z

[14] Sakaguchi S, Mikami N, Wing JB, Tanaka A, Ichiyama K, Ohkura N. Regulatory T cells and human disease. Annu Rev Immunol. 2020;38:541–66. doi: 10.1146/annurev-immunol-042718-041717 32017635

[15] Wang J, Gong R, Zhao C, Lei K, Sun X, Ren H. Human FOXP3 and tumour microenvironment. Immunology. 2023;168(2):248–55. doi: 10.1111/imm.13520 35689826

[16] Knochelmann HM, Dwyer CJ, Bailey SR, Amaya SM, Elston DM, Mazza-McCrann JM, et al. When worlds collide: Th17 and Treg cells in cancer and autoimmunity. Cell Mol Immunol. 2018;15(5):458–69. doi: 10.1038/s41423-018-0004-4 29563615 PMC6068176

[17] Bai Z, Zhou Y, Ye Z, Xiong J, Lan H, Wang F. Tumor-infiltrating lymphocytes in colorectal cancer: the fundamental indication and application on immunotherapy. Front Immunol. 2021;12:808964. 35095898 10.3389/fimmu.2021.808964 PMC8795622

[18] Syed Khaja AS, Toor SM, El Salhat H, Ali BR, Elkord E. Intratumoral FoxP3+Helios+ regulatory T cells upregulating immunosuppressive molecules are expanded in human colorectal cancer. Front Immunol. 2017;8:619. doi: 10.3389/fimmu.2017.00619 28603527 PMC5445103

[19] Goda N, Sasada S, Shigematsu H, Masumoto N, Arihiro K, Nishikawa H, et al. The ratio of CD8 + lymphocytes to tumor-infiltrating suppressive FOXP3 + effector regulatory T cells is associated with treatment response in invasive breast cancer. Discov Oncol. 2022;13(1):27. doi: 10.1007/s12672-022-00482-5 35438346 PMC9018954

[20] Baras AS, Drake C, Liu J-J, Gandhi N, Kates M, Hoque MO, et al. The ratio of CD8 to Treg tumor-infiltrating lymphocytes is associated with response to cisplatin-based neoadjuvant chemotherapy in patients with muscle invasive urothelial carcinoma of the bladder. Oncoimmunology. 2016;5(5):e1134412. doi: 10.1080/2162402X.2015.1134412 27467953 PMC4910705

[21] Fu H, Ward EJ, Marelli-Berg FM. Mechanisms of T cell organotropism. Cell Mol Life Sci. 2016;73(16):3009–33. doi: 10.1007/s00018-016-2211-4 27038487 PMC4951510

[22] Hughes CE, Nibbs RJB. A guide to chemokines and their receptors. FEBS J. 2018;285(16):2944–71. doi: 10.1111/febs.14466 29637711 PMC6120486

[23] Braoudaki M, Ahmad MS, Mustafov D, Seriah S, Siddiqui MN, Siddiqui SS. Chemokines and chemokine receptors in colorectal cancer; multifarious roles and clinical impact. Semin Cancer Biol. 2022;86(Pt 2):436–49. doi: 10.1016/j.semcancer.2022.06.002 35700938

[24] Koizumi K, Hojo S, Akashi T, Yasumoto K, Saiki I. Chemokine receptors in cancer metastasis and cancer cell-derived chemokines in host immune response. Cancer Sci. 2007;98(11):1652–8. doi: 10.1111/j.1349-7006.2007.00606.x 17894551 PMC11159633

[25] Hernández-Ruiz M, Zlotnik A. Mucosal chemokines. J Interferon Cytokine Res. 2017;37(2):62–70. 28207301 10.1089/jir.2016.0076 PMC5314990

[26] Koenecke C, Förster R. CCR9 and inflammatory bowel disease. Expert Opin Ther Targets. 2009;13(3):297–306. doi: 10.1517/14728220902762928 19236152

[27] Papadakis KA, Prehn J, Moreno ST, Cheng L, Kouroumalis EA, Deem R, et al. CCR9-positive lymphocytes and thymus-expressed chemokine distinguish small bowel from colonic Crohn’s disease. Gastroenterology. 2001;121(2):246–54. doi: 10.1053/gast.2001.27154 11487533

[28] Wurbel M-A, McIntire MG, Dwyer P, Fiebiger E. CCL25/CCR9 interactions regulate large intestinal inflammation in a murine model of acute colitis. PLoS One. 2011;6(1):e16442. doi: 10.1371/journal.pone.0016442 21283540 PMC3026821

[29] Löfroos A-B, Kadivar M, Resic Lindehammer S, Marsal J. Colorectal cancer-infiltrating T lymphocytes display a distinct chemokine receptor expression profile. Eur J Med Res. 2017;22(1):40. doi: 10.1186/s40001-017-0283-8 29020986 PMC5637168

[30] Wurbel MA, Malissen M, Guy-Grand D, Meffre E, Nussenzweig MC, Richelme M, et al. Mice lacking the CCR9 CC-chemokine receptor show a mild impairment of early T- and B-cell development and a reduction in T-cell receptor gammadelta(+) gut intraepithelial lymphocytes. Blood. 2001;98(9):2626–32. doi: 10.1182/blood.v98.9.2626 11675330

[31] Franco NH, Correia-Neves M, Olsson IAS. How “humane” is your endpoint? Refining the science-driven approach for termination of animal studies of chronic infection. PLoS Pathog. 2012;8(1):e1002399. doi: 10.1371/journal.ppat.1002399 22275862 PMC3261900

[32] Pérez-Cano FJ, Castellote C, González-Castro AM, Pelegrí C, Castell M, Franch A. Developmental changes in intraepithelial T lymphocytes and NK cells in the small intestine of neonatal rats. Pediatr Res. 2005;58(5):885–91. doi: 10.1203/01.pdr.0000182187.88505.49 16257927

[33] Wang H-C, Zhou Q, Dragoo J, Klein JR. Most murine CD8+ intestinal intraepithelial lymphocytes are partially but not fully activated T cells. J Immunol. 2002;169(9):4717–22. doi: 10.4049/jimmunol.169.9.4717 12391179

[34] Katoh H, Wang D, Daikoku T, Sun H, Dey SK, Dubois RN. CXCR2-expressing myeloid-derived suppressor cells are essential to promote colitis-associated tumorigenesis. Cancer Cell. 2013;24(5):631–44. doi: 10.1016/j.ccr.2013.10.009 24229710 PMC3928012

[35] Zhang X, Wei L, Wang J, Qin Z, Wang J, Lu Y, et al. Suppression colitis and colitis-associated colon cancer by anti-S100a9 antibody in mice. Front Immunol. 2017;8:1774. doi: 10.3389/fimmu.2017.01774 29326691 PMC5733461

[36] Karki R, Man SM, Malireddi RKS, Kesavardhana S, Zhu Q, Burton AR, et al. NLRC3 is an inhibitory sensor of PI3K-mTOR pathways in cancer. Nature. 2016;540(7634):583–7. doi: 10.1038/nature20597 27951586 PMC5468516

[37] Boivin GP, Washington K, Yang K, Ward JM, Pretlow TP, Russell R, et al. Pathology of mouse models of intestinal cancer: consensus report and recommendations. Gastroenterology. 2003;124(3):762–77. doi: 10.1053/gast.2003.50094 12612914

[38] Dzhalilova D, Zolotova N, Fokichev N, Makarova O. Murine models of colorectal cancer: the azoxymethane (AOM)/dextran sulfate sodium (DSS) model of colitis-associated cancer. PeerJ. 2023;11:e16159. doi: 10.7717/peerj.16159 37927787 PMC10624171

[39] Lee J-H, Jeon Y-D, Xin M, Lim J-Y, Lee Y-M, Kim D-K. Mast cell modulates tumorigenesis caused by repeated bowel inflammation condition in azoxymethane/dextran sodium sulfate-induced colon cancer mouse model. Biochem Biophys Rep. 2022;30:101253. doi: 10.1016/j.bbrep.2022.101253 35378739 PMC8976097

[40] Zuo B-W, Yao W-X, Fang M-D, Ren J, Tu L-L, Fan R-J, et al. Boris knockout eliminates AOM/DSS-induced in situ colorectal cancer by suppressing DNA damage repair and inflammation. Cancer Sci. 2023;114(5):1972–85. doi: 10.1111/cas.15732 36692143 PMC10154901

[41] Ostroumov D, Fekete-Drimusz N, Saborowski M, Kühnel F, Woller N. CD4 and CD8 T lymphocyte interplay in controlling tumor growth. Cell Mol Life Sci. 2018;75(4):689–713. doi: 10.1007/s00018-017-2686-7 29032503 PMC5769828

[42] De Simone M, Arrigoni A, Rossetti G, Gruarin P, Ranzani V, Politano C, et al. Transcriptional landscape of human tissue lymphocytes unveils uniqueness of tumor-infiltrating T regulatory cells. Immunity. 2016;45(5):1135–47. doi: 10.1016/j.immuni.2016.10.021 27851914 PMC5119953

[43] Creyns B, Cremer J, Hoshino T, Geboes K, de Hertogh G, Ferrante M, et al. Fibrogenesis in chronic DSS colitis is not influenced by neutralisation of regulatory T cells, of major T helper cytokines or absence of IL-13. Sci Rep. 2019;9(1):10064. doi: 10.1038/s41598-019-46472-6 31296924 PMC6624199

[44] Wang L, Ray A, Jiang X, Wang J, Basu S, Liu X, et al. T regulatory cells and B cells cooperate to form a regulatory loop that maintains gut homeostasis and suppresses dextran sulfate sodium-induced colitis. Mucosal Immunol. 2015;8(6):1297–312. doi: 10.1038/mi.2015.20 25807185 PMC4583327

[45] Pastille E, Bardini K, Fleissner D, Adamczyk A, Frede A, Wadwa M, et al. Transient ablation of regulatory T cells improves antitumor immunity in colitis-associated colon cancer. Cancer Res. 2014;74(16):4258–69. doi: 10.1158/0008-5472.CAN-13-3065 24906621

[46] Ling A, Edin S, Wikberg ML, Öberg Å, Palmqvist R. The intratumoural subsite and relation of CD8(+) and FOXP3(+) T lymphocytes in colorectal cancer provide important prognostic clues. Br J Cancer. 2014;110(10):2551–9. doi: 10.1038/bjc.2014.161 24675384 PMC4021513

[47] Grivennikov SI, Greten FR, Karin M. Immunity, inflammation, and cancer. Cell. 2010;140(6):883–99. doi: 10.1016/j.cell.2010.01.025 20303878 PMC2866629

[48] Bekker P, Ebsworth K, Walters MJ, Berahovich RD, Ertl LS, Charvat TT, et al. CCR9 antagonists in the treatment of ulcerative colitis. Mediators Inflamm. 2015;2015:628340. doi: 10.1155/2015/628340 26457007 PMC4592714

[49] Igaki K, Komoike Y, Nakamura Y, Watanabe T, Yamasaki M, Fleming P, et al. MLN3126, an antagonist of the chemokine receptor CCR9, ameliorates inflammation in a T cell mediated mouse colitis model. Int Immunopharmacol. 2018;60:160–9. doi: 10.1016/j.intimp.2018.04.049 29730559

[50] Trivedi PJ, Bruns T, Ward S, Mai M, Schmidt C, Hirschfield GM, et al. Intestinal CCL25 expression is increased in colitis and correlates with inflammatory activity. J Autoimmun. 2016;68:98–104. doi: 10.1016/j.jaut.2016.01.001 26873648 PMC4803021

[51] Wurbel M-A, Le Bras S, Ibourk M, Pardo M, McIntire MG, Coco D, et al. CCL25/CCR9 interactions are not essential for colitis development but are required for innate immune cell protection from chronic experimental murine colitis. Inflamm Bowel Dis. 2014;20(7):1165–76. doi: 10.1097/MIB.0000000000000059 24874458 PMC6249688

[52] Tsuchiya T. Role of gamma delta T cells in the inflammatory response of experimental colitis mice. J Immunol. 2003;171(10):5507–13. 14607957 10.4049/jimmunol.171.10.5507

[53] Melgar S, Karlsson A, Michaëlsson E. Acute colitis induced by dextran sulfate sodium progresses to chronicity in C57BL/6 but not in BALB/c mice: correlation between symptoms and inflammation. Am J Physiol Gastrointest Liver Physiol. 2005;288(6):G1328-38. doi: 10.1152/ajpgi.00467.2004 15637179

[54] Camuesco D, Rodríguez-Cabezas ME, Garrido-Mesa N, Cueto-Sola M, Bailón E, Comalada M, et al. The intestinal anti-inflammatory effect of dersalazine sodium is related to a down-regulation in IL-17 production in experimental models of rodent colitis. Br J Pharmacol. 2012;165(3):729–40. doi: 10.1111/j.1476-5381.2011.01598.x 21790535 PMC3315044

[55] Pabst O, Ohl L, Wendland M, Wurbel M-A, Kremmer E, Malissen B, et al. Chemokine receptor CCR9 contributes to the localization of plasma cells to the small intestine. J Exp Med. 2004;199(3):411–6. doi: 10.1084/jem.20030996 14744993 PMC2211800

[56] Fantini MC, Favale A, Onali S, Facciotti F. Tumor infiltrating regulatory T cells in sporadic and colitis-associated colorectal cancer: the red little riding hood and the wolf. Int J Mol Sci. 2020;21(18). 10.3390/ijms21186744 PMC7555219 32937953

[57] Evans-Marin HL, Cao AT, Yao S, Chen F, He C, Liu H, et al. Unexpected regulatory role of CCR9 in regulatory T cell development. PLoS One. 2015;10(7):e0134100. doi: 10.1371/journal.pone.0134100 26230654 PMC4521878

[58] Korbecki J, Grochans S, Gutowska I, Barczak K, Baranowska-Bosiacka I. Chemokines in a tumor: a review of pro-cancer and anti-cancer properties of receptors CCR5, CCR6, CCR7, CCR8, CCR9, and CCR10 ligands. Int J Mol Sci. 2020;21(20). 10.3390/ijms21207619 PMC7590012 33076281

[59] Liu J, Zhang N, Li Q, Zhang W, Ke F, Leng Q, et al. Tumor-associated macrophages recruit CCR6+ regulatory T cells and promote the development of colorectal cancer via enhancing CCL20 production in mice. PLoS One. 2011;6(4):e19495. doi: 10.1371/journal.pone.0019495 21559338 PMC3084880

[60] Svensson H, Olofsson V, Lundin S, Yakkala C, Björck S, Börjesson L, et al. Accumulation of CCR4⁺CTLA-4 FOXP3⁺CD25(hi) regulatory T cells in colon adenocarcinomas correlate to reduced activation of conventional T cells. PLoS One. 2012;7(2):e30695. doi: 10.1371/journal.pone.0030695 22319577 PMC3271060

[61] Ward ST, Li KK, Hepburn E, Weston CJ, Curbishley SM, Reynolds GM, et al. The effects of CCR5 inhibition on regulatory T-cell recruitment to colorectal cancer. Br J Cancer. 2015;112(2):319–28. doi: 10.1038/bjc.2014.572 25405854 PMC4301825

[62] Santagata S, Rea G, Bello AM, Capiluongo A, Napolitano M, Desicato S, et al. Targeting CXCR4 impaired T regulatory function through PTEN in renal cancer patients. Br J Cancer. 2024;130(12):2016–26. doi: 10.1038/s41416-024-02702-x 38704478 PMC11183124

[63] Brown JM, Wilson WR. Exploiting tumour hypoxia in cancer treatment. Nat Rev Cancer. 2004;4(6):437–47. doi: 10.1038/nrc1367 15170446

[64] Sarkar T, Dhar S, Sa G. Tumor-infiltrating T-regulatory cells adapt to altered metabolism to promote tumor-immune escape. Curr Res Immunol. 2021;2:132–41. doi: 10.1016/j.crimmu.2021.08.002 35492399 PMC9040151

[65] Villarreal DO, L’Huillier A, Armington S, Mottershead C, Filippova EV, Coder BD, et al. Targeting CCR8 induces protective antitumor immunity and enhances vaccine-induced responses in colon cancer. Cancer Res. 2018;78(18):5340–8. doi: 10.1158/0008-5472.CAN-18-1119 30026324

[66] Zhou B, Lawrence T, Liang Y. The role of plasmacytoid dendritic cells in cancers. Front Immunol. 2021;12:749190. doi: 10.3389/fimmu.2021.749190 34737750 PMC8560733

[67] Mishalian I, Bayuh R, Eruslanov E, Michaeli J, Levy L, Zolotarov L, et al. Neutrophils recruit regulatory T-cells into tumors via secretion of CCL17--a new mechanism of impaired antitumor immunity. Int J Cancer. 2014;135(5):1178–86. doi: 10.1002/ijc.28770 24501019

[68] Sun W, Wei F-Q, Li W-J, Wei J-W, Zhong H, Wen Y-H, et al. A positive-feedback loop between tumour infiltrating activated Treg cells and type 2-skewed macrophages is essential for progression of laryngeal squamous cell carcinoma. Br J Cancer. 2017;117(11):1631–43. doi: 10.1038/bjc.2017.329 28949956 PMC5729431

