# Supplementary figures and images for: CCR9 shapes the immune microenvironment of colorectal cancer modulating the balance between intratumoral CD8+ T cell and FoxP3+ Helios+ Treg subpopulations
Source: PLoS One. 2025 Apr 30;20(4):e0321930. doi: 10.1371/journal.pone.0321930 (PMC12043142; doi:10.1371/journal.pone.0321930)

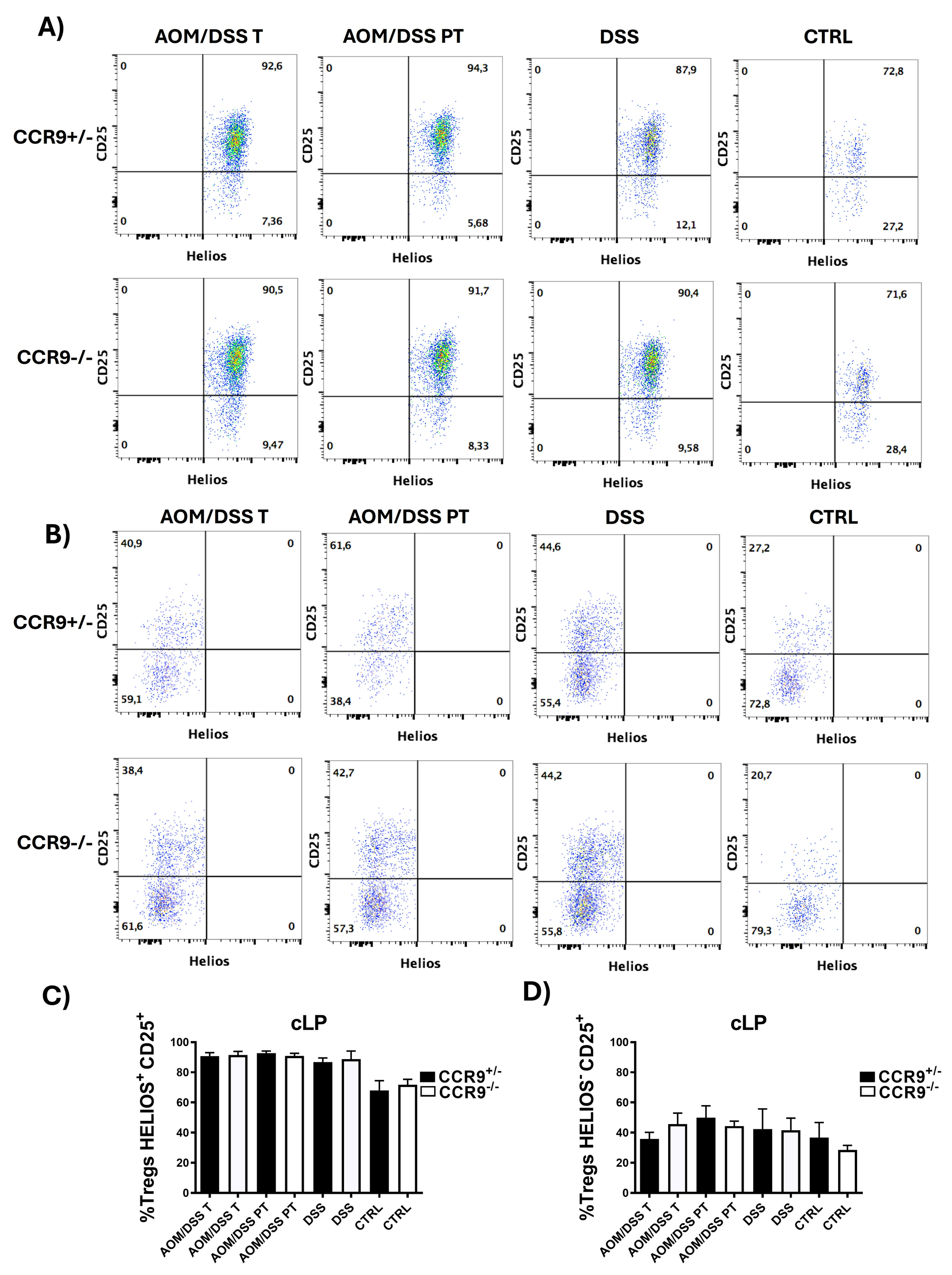

Supplement: S1 Fig — (TIF) [file pone.0321930.s001.tif]
